# Urinary ACE Phenotyping as a Research and Diagnostic Tool: Identification of Sex-Dependent ACE Immunoreactivity

**DOI:** 10.3390/biomedicines11030953

**Published:** 2023-03-20

**Authors:** Alexander J. Kozuch, Pavel A. Petukhov, Miklos Fagyas, Isolda A. Popova, Matthew O. Lindeblad, Alexander P. Bobkov, Armais A. Kamalov, Attila Toth, Steven M. Dudek, Sergei M. Danilov

**Affiliations:** 1 Department of Medicine, Division of Pulmonary, Critical Care, Sleep and Allergy, University of Illinois at Chicago, CSB 915, MC 719, 840 S. Wood Ave., Chicago, IL 60612, USA; 2 Department of Pharmaceutical Sciences, College of Pharmacy, University of Illinois at Chicago, 833 S Wood St, Chicago, IL 60612, USA; 3 Division of Clinical Physiology, Department of Cardiology, University of Debrecen, Nagyerdei krt. 94, 4032 Debrecen, Hungary; 4 Toxicology Research Laboratory, University of Illinois at Chicago, 840 S. Wood Ave., Chicago, IL 60612, USA; 5 Faculty of Fundamental Medicine, Moscow University, Moscow 117192, Russia; 6 Medical Center, Moscow University, Moscow 119435, Russia

**Keywords:** angiotensin I-converting enzyme, human urine, sex-dependent differences, outliers, conformational changes, glycosylation, screening

## Abstract

Background: Angiotensin-converting enzyme (ACE) is highly expressed in renal proximal tubules, but ACE activity/levels in the urine are at least 100-fold lower than in the blood. Decreased proximal tubular ACE has been associated with renal tubular damage in both animal models and clinical studies. Because ACE is shed into urine primarily from proximal tubule epithelial cells, its urinary ACE measurement may be useful as an index of tubular damage. Objective and Methodology: We applied our novel approach—ACE phenotyping—to characterize urinary ACE in volunteer subjects. ACE phenotyping includes (1) determination of ACE activity using two substrates (ZPHL and HHL); (2) calculation of the ratio of hydrolysis of the two substrates (ZPHL/HHL ratio); (3) quantification of ACE immunoreactive protein levels; and (4) fine mapping of local ACE conformation with mAbs to ACE. Principal findings: In normal volunteers, urinary ACE activity was 140-fold less than in corresponding plasma/serum samples and did not differ between males and females. However, urinary ACE immunoreactivity (normalized binding of 25 mAbs to different epitopes) was strongly sex-dependent for the several mAbs tested, an observation likely explained by differences in tissue ACE glycosylation/sialylation between males and females. Urinary ACE phenotyping also allowed the identification of ACE outliers. In addition, daily variability of urinary ACE has potential utility as a feedback marker for dieting individuals pursuing weight loss. Conclusions/Significance: Urinary ACE phenotyping is a promising new approach with potential clinical significance to advance precision medicine screening techniques.

## 1. Introduction

Angiotensin I-converting enzyme (ACE, CD143) is a Zn^2+^ carboxydipeptidase that plays key roles in the regulation of blood pressure and in the development of vascular pathology. ACE is constitutively expressed on the surface of endothelial cells, absorptive epithelial and neuroepithelial cells, and cells of the immune system (macrophages, dendritic cells) [1,2]. Circulating blood ACE originates from endothelial cells [3], primarily lung capillary endothelium [4] by proteolytic cleavage [5]. In healthy individuals, blood ACE levels remain very stable throughout an individual’s lifetime [6], whereas in granulomatous diseases (e.g., sarcoidosis) and Gaucher’s disease, blood ACE activity is significantly increased [7].

Especially high expression of ACE (even higher than in lung endothelial cells) was found within the brush border of the proximal tubular cells of the kidney [8,9,10]. ACE was found in the tubular fluid along the whole nephron but contained significantly different amounts of ACE [11], which was also species-specific [10]. It is also necessary to mention that ACEs in different compartments of tubular fluid are different: in the peripheral blood, in the blood of glomerular efferent or afferent arterioles, and in capillaries ACE originates (sheds) from capillary endothelial ACE, whereas ACE in tubular fluid originates mainly from proximal tubules epithelial cells. Moreover, local ACE conformation from endothelial cells and from epithelial cells is different, which could be distinguished by a set of mAbs to different epitopes of ACE [12,13].

ACE has a high molecular mass (170 kD) and is not permeable during glomerular filtration. Thus, the ACE activity of urine [14,15] likely derives mainly from proximal tubular cells, suggesting the potential usefulness of urinary ACE measurement as an index of tubular damage [16]. The COVID-19 pandemic has provided an additional stimulus for improved detection of renal tubular damage. The kidney is a key organ affected by SARS-CoV-2 [17], and a significant proportion of autopsy specimens have moderate to severe renal tubular damage in fatal COVID-19 infection [18]. However, studies of urinary ACE are limited. This is in part because its activity in urine is very low, the accuracy of the present standard assays is not proven, and no reliability criteria have been specified [19]. In addition, mammalian urine contains naturally occurring angiotensin-converting enzyme inhibitors [15]. Nevertheless, prior studies have demonstrated a threefold increase in urinary ACE in patients with upper urinary tract infections and renal calculi [16] and a three- to fivefold increase in patients with chronic glomerulonephritis and nephrotic syndrome [20]. Elevated ACE excretion into urine was also found in diabetic patients with overt nephropathy [21], and even different fragments of the N domain of ACE were found [22].

We recently developed a novel approach to characterize ACE in tissue and blood—ACE phenotyping—which may be useful for improved characterization of urinary ACE in patients with nephropathy. This ACE phenotyping approach includes only (1) determination of ACE activity (with two substrates (ZPHL and HHL); (2) calculation of a novel kinetic parameter-ratio of hydrolysis of these two substrates (ZPHL/HHL ratio) [23]; (3) quantification of ACE immunoreactive protein levels [24]; and (4) ACE conformation mapping with a unique set of mAbs to ACE—reviewed in [13].

In the first stage of this project, we applied ACE phenotyping to characterize urinary ACE in healthy volunteers. Unexpectedly, we found dramatic differences in immunoreactivity of male and female urinary ACEs that may be explained by sex-specific differences in kidney ACE glycosylation and/or sialylation. In addition, this ACE phenotyping approach was able to identify ACE outliers using only urine. Thus, urinary ACE phenotyping is a promising new approach with potential clinical significance to advance precision medicine screening techniques, specifically as a new research and diagnostic tool to study renal tubular damage.

## 2. Materials and Methods

### 2.1. Chemicals 

ACE substrates, benzyloxycarbonyl-L-phenylalanyl-L-histidyl-L-leucine (Z-Phe-His-Leu) and hippuryl-L-histidyl-L-leucine (Hip-His-Leu) were purchased from Bachem Bioscience Inc. (King of Prussia, PA, USA). Other reagents (unless otherwise indicated) were obtained from Sigma-Aldrich (St. Louis, MO, USA). AM-15 and AM-4 ultrafiltration membranes (cut-off Mr 3000 and 100,000, respectively, were from Merck Millipore Ltd. (Cork, Ireland), and dialysis cassettes (cut-off 10,000) were from Thermo Scientific (Rockford, IL, USA).

### 2.2. Antibodies 

Antibodies used in this study include a set of 25 mAbs to human ACE, recognizing native conformation of the N and C domains of human ACE [25,26].

### 2.3. Study Participants 

The collection of human samples was approved by the Ethics Committee of the University of Debrecen (Hungary) as described in detail previously [27]. All corresponding procedures were carried out in accordance with institutional guidelines and the Code of Ethics of the World Medical Association (Declaration of Helsinki). All volunteers and patients provided written informed consent to have different human tissue samples for ACE characterization.

### 2.4. Urines

Urine was from 11 apparently healthy volunteers (primarily laboratory staff) (5 men, mean age 40.2 ± 20.1 years, and 6 women, mean age 41.0 ± 19.7 years) without any known renal disorder, as judged by a normal diuresis and normal protein content (less than 30 ug/mL). One volunteer (male, 51 y.o.) has had diabetes mellitus (type 2) since 2013 without nephropathy. 

Freshly collected morning urine (200 mL) from the volunteers was centrifuged at 2000 g for 3 min (to collect cells shed into the urine). Then, the pH of the supernatant (urine without cell pellet and debris) was measured (the range of the pH was from 5.3 to 6.6) and adjusted to neutral values (7.4–8.0) using 1M Tris-HCl, pH 8.0 or 1.4M NaOH, in order to minimize the action of the proteolytic enzymes), followed by concentrating the samples 30-fold (using filtration of urine by centrifugation at 4000× *g*) on a filter (Amicon-15, Millipore) with 3 kD pores. Next, 0.5 mL of 30X concentrated urine samples were dialyzed overnight (at +4C) against PBS with 10 μM of Zn^2+^ (for determination of ACE activity with two substrates), and the remainder was used for immunological characterization (ACE activity precipitation by numerous mAbs to ACE).

In some experiments 30-fold-concentrated urine (5 mL) was further concentrated another 10-fold using AM-4 filters with pores 100 kD, and then the volume (0.5 mL) was restored to the initial level (5 mL). Thus, we decreased the concentration of all proteins in the urine (with molecular weight up to 100 kD) by 10-fold (100–kD depletion experiments).

Tissue processing (lung, heart, and lymph nodes from 9 donors for each tissue) for further determination of ACE activity and immunoreactive ACE protein was performed as described in detail previously [27].

### 2.5. ACE Activity Assay

ACE activity in serum/plasma was measured using a fluorimetric assay with two ACE substrates, 2 mM Z-Phe-His-Leu or 5 mM Hip-His-Leu [28]. Calculation of the ZPHL/HHL ratio [23] was performed by dividing the fluorescence of the reaction product produced by the ACE sample with ZPHL as a substrate by that with HHL as a substrate. 

### 2.6. Immunological Characterization of the Blood ACE 

Another approach for the quantification of ACE levels, even in the presence of ACE inhibitors, EDTA, or pigments/fluorochromes, is to use an immunoassay (in which ACE initially is captured from biological fluids by antibodies). Several of these immunoassays have been developed, including radioimmunoassay or variations of immunoassays with polyclonal and/or monoclonal antibodies. However, these immunoassays have limited general utility [24]. 

In order to quantify the amount of immunoreactive ACE protein in the urine, we applied another version of immunoassay, in which native, catalytically active ACE from urine samples was captured by anti-ACE mAbs that recognize conformational epitopes on the surface of ACE molecules. Next, after washing away the unbound ACE (and all components of urine, including possible ACE inhibitors and pigments), precipitated ACE activity was quantified directly in the wells of microtiter plates by fluorometry after adding the substrates ZPHL or HHL [24,28].

Microtiter (96-well) plates (Corning, Corning, NY, USA) were coated with anti-ACE mAbs via goat anti-mouse IgG (Invitrogen, Rockford, IL, USA) bridge and incubated with plasma/serum/lung ACE samples. After washing unbound ACE, the level of ACE immunoreactive protein was quantified as described previously using the strong mAb 9B9 [24,28]. Conformational fingerprinting of ACE was performed as described using a set of mAbs to different epitopes of ACE [25].

### 2.7. Whole Exome Sequencing

Genomic DNA was obtained from cell pellets after centrifugation (3000× *g*) of urine of tested subjects (#1A and #9Q), or from saliva using a QIAamp DNA Mini Kit (Qiagen, Valencia, CA, USA). Whole exome sequencing (WES) and bioinformatic analysis of the sequencing data were carried out by Novogene (Sacramento, CA, USA). 

### 2.8. Computational Analysis of the Dimer Model with Human Serum Albumin (HSA)

The dimer model of somatic ACE was prepared as described previously [29]. The missing portion of the ACE protein between the C and N domains was reconstructed using the Linker Modeler module in a Molecular Operating Environment (MOE, https://www.chemcomp.com/index.htm, accessed on 1 May 2022). Asn residues in the ACE protein were glycosylated with N-acetylglucosamine in MOE. Human Serum Albumin (HSA) X-ray structure 1E78 [30] was downloaded from Protein Databank. The buffer components were removed from the HSA model. Both the ACE dimer and the HSA proteins were subjected to the “structure preparation” procedure using AMBER14EHT forcefield implemented in MOE [31,32]. Hydrogen atoms were added using the Protonate 3D algorithm in MOE.

For protein–protein docking, the ACE dimer and the HSA protein were used as a receptor and a ligand, respectively. The default settings were used for all the docking. The “Patch Analysis” option was used to account for the hydrophobic patch potential. Post-placement refinement was conducted using the “rigid body” option. One of the top models where the HSA protein is close to epitopes 1E10, 5F1/2D1, and 1B8/3F10 was selected for further analysis in PYMOL [33].

### 2.9. Statistical Analysis

Values of ACE activity with different substrates for each individual, as well as other parameters characterizing the ACE phenotype, were means ±SD from 2 to 5 independent experiments (depending on the individual) with triplicates. Significance was analyzed using the Mann–Whitney test.

## 3. Results

### 3.1. ACE Activity in Human Urine Samples

Despite the fact that ACE is highly expressed in renal proximal tubules [10], ACE activity/levels in the urine are 50–300-fold lower than in the blood (depending on the method of measurement [14,19,23,34]). In some studies, ACE activity in control urine was not detected at all [19]. Therefore, enzymatic activity measurement is difficult to perform without a prior concentration of urine and ultrafiltration/dialysis to remove endogenous ACE inhibitors [15,19] and pigments that interfere with the measurement of fluorescence [23]. 

Therefore, we collected urine samples (200 mL) from 16 volunteers (primarily laboratory staff), centrifuged them (to collect cells shed into the urine), and adjusted their pH to neutral values. They then were concentrated 30-fold (using filtration by centrifugation) on filters with 3 kD pores. Next, the concentrated urine samples were dialyzed overnight (at +4C) against PBS with 10 μM of Zn^2+^. ACE activity then was measured in 1/5 diluted samples (to decrease fluorescence quenching) using a fluorimetric assay with two artificial (short) substrates-ZPHL and HHL [23,28]. 

**Figure 1:**
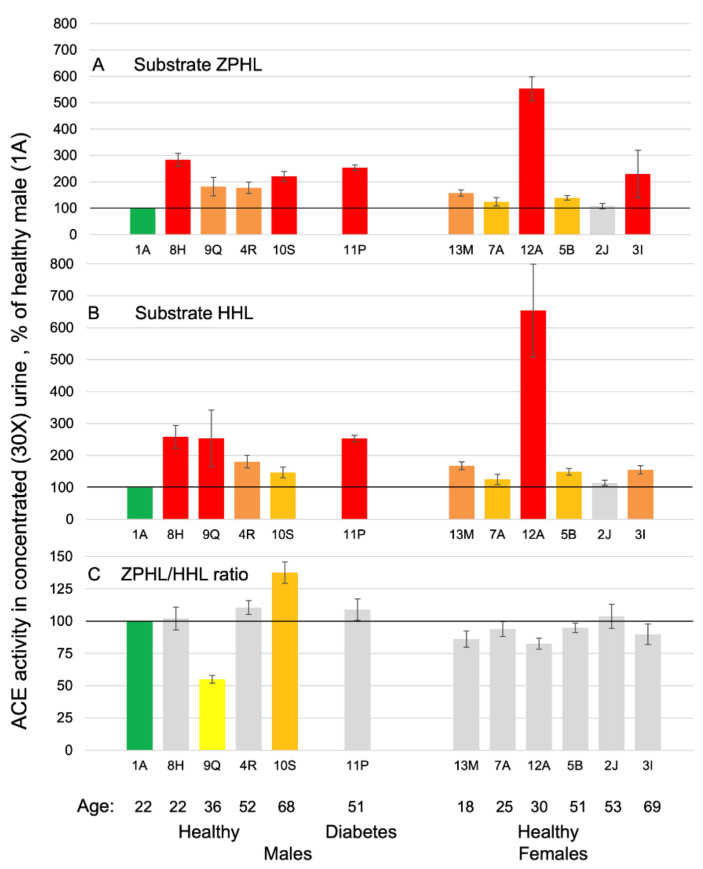
**ACE activity in human concentrated urine**. ACE activity in 12 samples of concentrated (30X) and dialyzed urine was quantified using a spectrofluorometric assay with Z-Phe-His-Leu (1 mM) (**A**) and Hip-His-Leu (2.5 mM) (**B**) as substrates (**C**). Ratio of the rate of hydrolysis of the two substrates (ZPHL/HHL ratio) in the tested samples. Data expressed as % of individual urinary ACE activity from the designated “gold standard” (1A-urine of healthy 22 y.o. male taken as 100%-green bar). Bars with significant changes in % of control ACE activity are colored as follows: increase of more than 20%—orange, >50%—brown, more than 2-fold—red, decrease of more than 20%—yellow, and more than 50%—light blue. Mean values (±SD) from 2 to 5 experiments (each made in triplicates).

Urinary ACE activity in our cohort of five apparently healthy males and six females (Figure 1) varied by sixfold for both substrates.

These results are similar to the three- to sixfold differences in urine ACE activity previously reported in early studies in apparently healthy subjects [14,34] and they are more variable than serum ACE activity in multiple previous reports (threefold differences) [6,24,28]. Other prior studies have reported three- to fivefold increases in urinary ACE activity in patients with upper urinary tract infections and renal calculi [19] in patients with chronic glomerulonephritis and nephrotic syndrome [20] and in diabetic patients with nephropathy [21]. Because of this huge range in normal values of urinary ACE activity, and an even greater range in patients with different renal pathologies [34], measurement of urinary ACE activity has been of limited clinical significance and primarily used only for research purposes to characterize differences between various groups of patients.

As in early studies [14,19], we did not find significant differences in urinary ACE activity between men and women (Figure 1A,B). This observation supports the interpretation that urinary ACE is derived mainly from kidney tubular cells, without any urinary contamination from gonadic and prostatic ACE. These data confirm previous conclusions [19]. No sex-specific differences in ACE activity were previously reported in human lung and heart tissues [27] or in human plasma/serum in numerous studies [35,36]. 

Comparison of the absolute values of ACE activity in the urine samples (Figure 1A,B) with the ACE activity in available plasma from four volunteers (Figure S1A,B) demonstrated that urinary ACE activity is approximately 140-fold less than in the plasma/serum. Because ACE expression in the kidney proximal tubule epithelial cells is very high [8,9,10] and comparable with the level of ACE expression in lung endothelial cells in humans (and even several-fold higher in some species [37]), we may hypothesize that very low ACE activity in the human urine may be explained by (1) a significant decrease in the expression of an unidentified ACE secretase in the proximal tubules epithelial cells in comparison to that in endothelial cells, and/or by (2) the presence of strong inhibitors of ACE shedding precisely in the proximal tubule epithelial cells. Promising candidates for such inhibitors of ACE shedding include lysozyme and bilirubin, which both have very high local concentrations in the proximal tubules due to the effective glomerular filtration and tubular reabsorption of these substances [38,39]. The binding of these compounds to ACE fixes its conformation and prevent excessive ACE shedding [40].

We next calculated an important catalytic parameter of ACE-the ratio of the rates of hydrolysis of two ACE substrates, ZPHL and HHL (ZPHL/HHL ratio [23] and found that this ratio was substantially decreased in urinary sample #9Q (Figure 1C). The two domains of ACE hydrolyze a range of natural and synthetic substrates with different efficiencies [23,41]. Two synthetic substrates, ZPHL and HHL, are used for the determination of ACE activity in laboratories worldwide. These two substrates display some contrasting enzymatic properties: the C domain of human ACE hydrolyzes HHL at a much faster rate (ninefold) than the N domain, whereas ZPHL is hydrolyzed at a similar rate by both domains. As a result, their ratio is characteristic of different forms of ACE [23,41]. The selective inactivation or inhibition of the C-domain in somatic ACE increases this ratio to higher values more characteristic for the N-domain, whereas selective inactivation/inhibition of the N-domain in somatic ACE decreases the ratio toward lower values predicted for the C-domain. The absolute value of the ZPHL/HHL ratio for urinary ACE in subject #9Q is 55% of that for the “gold standard” (urine sample from healthy male, 22 y.o. #1A—first author of this paper) (Figure 1C) and represents the lowest ZPHL/HHL ratio we have determined in more than 800 tested plasma/serum samples over many years. Therefore, this very low value of the ZPHL/HHL ratio for the urinary ACE in subject #9Q suggests that certain amino acid residues either close to or in the N domain active center of ACE may be altered by a mutation.

Previously, we described another patient with a similar, but less pronounced decrease in the ZPHL/HHL ratio (by 33 %), which carried an ACE mutation in the N domain of ACE-S333W [41]. This parameter, ZPHL/HHL ratio, was increased in the urine of male volunteer #10S, who was taking 10 mg of the ACE inhibitor lisinopril/day (Figure 1C and Figure S1C), as well as in another male volunteer-#14D (Figure S1C). This increase indicates that ACE in concentrated urine is still able to bind some low molecular weight ACE inhibitors, despite the dialysis that occurs during sample processing.

### 3.2. Immunoreactivity of ACE in Human Urine

**Figure 2:**
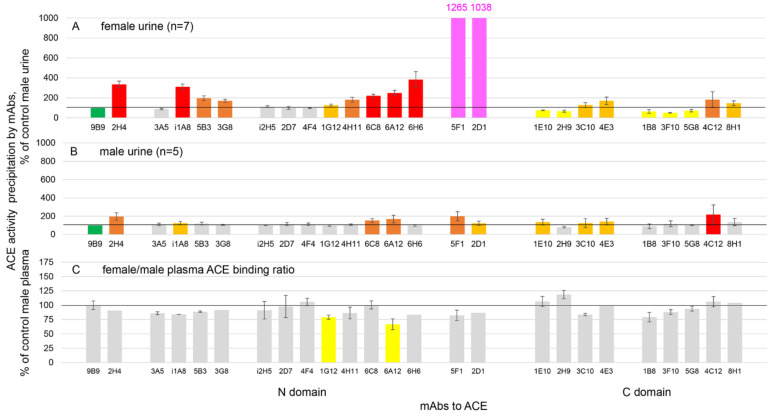
**Sex-dependence in urinary ACE immunoreactivity.** ACE activity was precipitated from 30X concentrated urine samples of 4 apparently healthy females (**A**) and 4 apparently healthy males (**B**) by 25 mAbs to different epitopes of human somatic ACE. (**C**). Ratio of ACE activity precipitation from citrated plasma of females to that from males by 25 tested mAbs. Data are expressed as % from ACE activity precipitated from sample #1A (urine from 22 y.o. healthy male) used as a control sample and normalized by binding by mAb 9B9 (mAbs/9B9 ratios). Mean values (±SD) from 2 to 5 experiments (each made in triplicates). Bars are colored as in Figure 1.

The levels of ACE immunoreactive protein were first determined in a pilot experiment using a set of mAbs to ACE to analyze urine samples from three females and one male, and the results were absolutely unexpected. Precipitation of urinary ACE (normalized for ACE activity) from all three female subjects was significantly higher than precipitation of urinary ACE from the male subject (55 y.o.) for 4 mAbs to the N domain (out of 8 mAbs tested), and it was significantly lower for all eight tested mAbs to the C domain (Figure S2). To further study this possible sex-specific difference in local ACE conformation, we next compared the pattern of mAbs binding to male and female urinary ACE using the entire set of 25 mAbs to the epitopes on the N and C domains [26] of somatic ACE (Figure 2A,B). The binding of 20 mAbs (out of the 25 tested) to urinary ACE from seven apparently healthy women (from 18 to 69 y.o.) was dramatically different in comparison to the “gold standard” of urinary ACE from the 22 y.o. healthy male (Figure 2A-green bars), confirming the previous results obtained from another male (Figure S2).

In the series of papers from Casarini’s lab, the presence of the N domain fragment(s) of ACE in the concentrated human urine was demonstrated [11], similar to the N domain fragment found naturally in the ileal fluid [42] or after treatment of purified ACE with exogenous proteases [43] or in concentrated urine by endogenous proteases [23]. However, our results with preferential binding of some mAbs to female urinary ACE could not be explained by the presence of N fragment(s) in the female urine, because in that case an increase in mAbs-binding would be seen for all mAbs directed to the epitopes on the N domain, whereas differences in mAbs binding were strongly epitope-specific but not domain-specific—Figure 2A. Moreover, experiments in Figure 2 were performed on urine concentrated 30-fold using a 3 kD filter, thus retaining putative N fragments.

One practical conclusion from Figure 2A is that the binding of several mAbs (3A5, i2H5/2D7, 4F4-grey bars) to urinary ACE was not gender-dependent. Therefore, these mAbs could be used for the quantification of urinary ACE levels regardless of gender. In contrast to the observations in female urine, the binding of these 25 mAbs was much more consistent for urinary ACE from five apparently healthy males from 22 to 68 y.o. (Figure 2B). Of note, the binding of mAbs to urinary ACEs in Figure 2 was normalized by ACE activity precipitation by the strongest mAb 9B9 [26,44,45], which avoids an additional determination of ACE activity in the tested urines, and thus significantly increases accuracy and reproducibility of the repeated determinations. Indeed, inter-individual variations of mAb binding exist (Figure 2B) because ACE mutations have been identified for almost every amino acid residue in ACE molecule (the NCBI SNP database for ACE https://www.ncbi.nlm.nih.gov/SNP/snp_ref.cgi?locusId = 1636, accessed on 1 May 2022). These mutations may influence some mAb binding in some patients [26,46], but the gender differences in mAb binding to urinary ACE still appear to be very significant (Figure 2A,B).

Especially impressive sex differences in mAbs binding (i.e., in local ACE conformation) were revealed when we compared the binding of mAb 2D1 (which has an epitope on the N domain) and mAb 2H9 (epitope on the C domain of ACE) [26] to urinary ACE from male and female samples (Figure S3A,B). The calculated parameter of 2H9/2D1 binding ratio was 10-fold higher for four urinary ACE samples from randomly selected males than for four urinary ACE samples from randomly selected females (Figure S3C). Using only the very limited number of the cells that could be sedimented from urine samples of apparently healthy males and females (including renal tubular epithelial cells that highly express ACE) [47,48], and the correspondingly limited ACE activity in these cell lysates, we demonstrated that differences in the binding of some mAbs to male and female ACEs (i.e., representing the local ACE conformation) are retained for membrane-bound ACE in these cell lysates (Figure S3D–F).

We previously demonstrated that changes in the mAb binding pattern to different ACEs were determined by alterations in ACE glycosylation/sialylation that occur in different organs/tissues—i.e., the concept of ACE conformational fingerprinting [13,49]. Interest in sex-dependent glycosylation of proteins initially derived from observations regarding sexual dimorphism in autoimmunity. The frequency and severity of various infectious diseases are higher in males than in females, suggesting that females may have a stronger immune response. The potential detrimental effect of this sex-dependent phenomenon is that females are more likely to develop many autoimmune diseases, such as systemic lupus erythematosus (SLE), Grave’s disease, Hashimoto’s thyroiditis, multiple sclerosis, rheumatoid arthritis and scleroderma [50]. One initial hypothesis was that differences in IgG glycosylation between males and females may contribute to sex dependence in autoimmune diseases. Later, it was observed that galactosylation of IgG is controlled by estrogen, and IgG-dependent inflammation differs in males and females [51]. Sex-associated differences in N-glycosylation were identified not only in IgG from whole serum, but also in IgG from human salivary glycoproteins [52], in whole murine [53], and human plasma proteins [54] in snake venom [55] and in insects [56]. Sex-specific differences in N glycosylation are also present in individual proteins such as AQP1 [57] and glycodelin [58]. However, the differential glycosylation of glycodelin in human amniotic fluid and in seminal fluid may be an example of tissue-specific glycosylation, rather than sex-specific, a similar effect as in prostate-specific glycosylation of ACE [13,49]. In contrast, the dramatic difference in the pattern of mAb binding (i.e., conformational changes likely reflecting glycosylation) in urinary ACE from males and females (Figure 2A,B and Figure S2) is an example of a pure sex-dependent effect because it occurs on ACEs originating from the same tissue (human kidney). Recently, 1016 *N*-glycoproteins and 2192 *N*-glycopeptides were identified in urine by liquid chromatography–mass spectrometry (LC-MS), and their abundance spanned across approximately five orders of magnitude. In females, 175 *N*-glycoproteins were significantly down-regulated (fold change >4) and 31 significantly up-regulated compared to males [59]. The sex-specific differences in urinary ACE that we identify here are rather qualitative (differences in glycosylation of certain glycosylation sites) and likely not related to differences in total ACE protein abundance.

**Figure 3:**
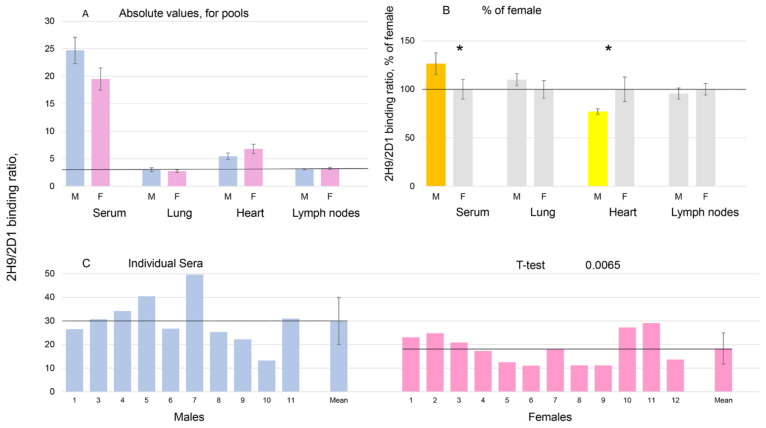
**The 2H9/2D1 binding ratio for ACE derived from several human tissues.** ACE activity in the indicated tissue homogenates, or sera from male and female patients, was approximately equilibrated according to their ACE activity. Then, ACE activity from these samples was precipitated using mAbs 2H9 and 2D1 (as in Figure 2). The 2H9/2D1 binding ratio for each tissue was expressed in absolute values (**A**), or as a % of mean from the female sample values. Shown are mean values (±SD) from 4 to 6 experiments (each made in triplicates). M—male; F—female. Bars colored in (**B**)—as in Figure 1. Results from 10 male sera and 12 female sera are shown in (**C**). This experiment was performed in triplicates. The SD is <10% and was not shown for clarity.* Values in colored bars of Figure 3B for males are statistically significant (*p* < 0.05) from females.

We also calculated the 2H9/2D1 binding ratio as a marker for the putative sex difference in ACE (Figure S3C) from ACE derived from different human tissues (Figure 3). This ratio (which likely indicates the extent of sialylation of ACE at position Asn45) was tissue specific. The highest ratio was found for serum ACE: approximately 20, which reflects the known high extent of sialylation of blood ACE [60]. The lowest ratios (~3) were observed for ACE derived from the lung and lymph nodes, whereas a higher ratio (~6) was present for heart ACE (Figure 3A). This ratio did not differ as dramatically between male and female ACE in these tissues (Figure 3B) as it did for urinary ACE, in which the 2H9/2D1 binding ratio was eightfold higher in males than in females (Figure S3). 

Nevertheless, some gender differences are evident in these tissues. The 2H9/2D1 ratio was significantly lower for ACE in male heart tissue than from female heart tissue, whereas the opposite pattern was observed for serum ACE in which male samples were higher than female samples (Figure 3B). In the 10 male and 12 female sera samples analyzed here, the increased 2H9/2D1 binding ratio observed in male sera was statistically significant (Figure 3C). However, the sex differences in these tissues (heart and serum) were less pronounced than for urinary ACE (which represents kidney ACE).

It is not surprising that these dramatic differences in mAb binding to female compared to male urinary ACE are almost completely absent in serum ACE (Figure 2C). Endothelial ACE that is shed from the cell surface into the blood almost immediately passes through the liver, where multiple glycoforms of ACE that are without terminal galactose or sufficient sialic acids are trapped by asialoglycoprotein receptors [61]. In contrast, ACE shed into urine from proximal epithelial cells does not leave the renal tubules [62,63] does not contact any biological filters, and thus retains the huge diversity of its glycoforms. Nevertheless, few mAbs to the N domain (6A12/1G12) can distinguish male and female ACE even in plasma (Figure 2C), which may indicate that not only sialylation, but also glycosylation itself may be responsible for differences in the binding of these mAbs.

Since the glycosylation of proteins in general, and ACE in particular (having 17 potential glycosylation sites [64]), is involved in proper protein folding, protein quality control, transport of proteins to specific organelles, and sensitivity to shedding [65,66,67], the sex-specific differences in tissue ACE glycosylation that we have identified may be associated with differential disease involvement. Recently, increased female susceptibility to Alzheimer’s disease was found in the carriers of the novel ACE mutation R1279Q [68] which may be explained by significant differences in the conformation of ACE in male and female tissue ACEs (Figure 2, Figures S2 and S3). Based on our data, the increased female susceptibility to Alzheimer’s disease in the carriers of ACE mutation R1279Q may be due to direct structural differences between male and female brain ACE (i.e., due to differences in glycosylation of male and female ACE), or indirectly due to functional differences in male and female brain ACE caused by differential interactions with putative ACE-binding proteins (or substrates) that result from structurally different male and female ACE in the brains of patients with this mutation. We also can not exclude the possibility that the prevalence of functional pain syndrome in women [69] partially may be explained by gender differences in tissue ACE that we identified, because the pain mediators substance P and bradykinin are substrates for ACE and are effectively cleaved by ACE [70,71]. Therefore, we speculate that subtle sex differences in the local conformation of ACE in brain neurons may contribute to the increased female prevalence of both disease processes. 

### 3.3. Characterization of Urinary ACE

**Figure 4:**
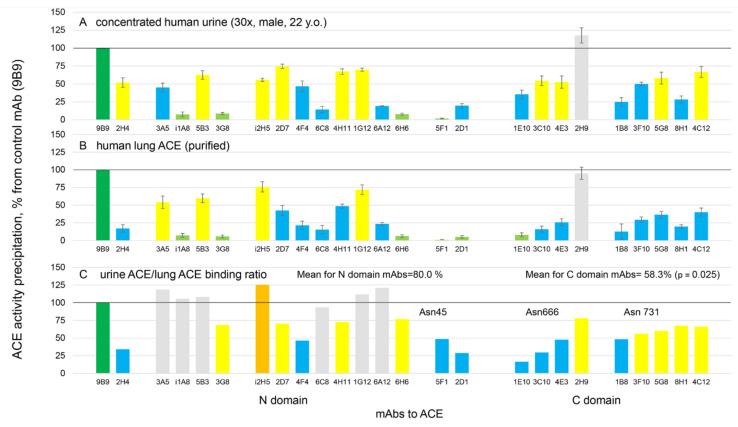
**Immunoreactivity of urinary ACEs and ACE purified from human lung.** ACE activity was precipitated from 30x concentrated urine (**A**) of 22 y.o. healthy male-sample #1A and ACE preparation purified from male human lung (**B**) by 25 mAbs to different epitopes of human somatic ACE. Data are expressed as % of ACE activity precipitated by tested mAbs to that by mAb 9B9 used as a control mAb (mean ± SD). (**C**) Ratio of ACE activity precipitation from male urine to that from ACE purified from male human lung. Values of standard deviations for ratios, which were not exceeded 10%, were not shown for clarity. Bars are colored as in Figure 1. In addition, mAbs in which binding to ACE was less than 10% of that for mAb 9B9 were marked with light green.

To further characterize urinary ACE, we compared the “conformational fingerprint” patterns of binding of 25 mAbs to different epitopes on the N and C domain [26] for our “gold standard” urinary ACE (from male subject #1A-) and human lung ACE purified from a male (Figure 4). The magnitude of the bars shown in Figure 4A,B reflect affinity binding of the individual mAbs to each type of ACE and are not as informative as the urine ACE/lung ACE binding ratio (Figure 4C), which clearly demonstrated significant differences in the local conformation between urinary ACE (in its relatively natural environment, albeit 30X concentrated) and lung ACE. It is readily apparent from this ratio that changes in mAb binding (i.e., in local ACE conformation) between urinary ACE and lung ACE are more pronounced in the C domain. We calculated the mean % change for mAbs to the N domain and the C domain, and the difference was significant (80% versus 58.3%, *p* = 0.025). Theoretically, differences in mAb binding to urinary ACE compared to pure lung ACE may be due to at least two reasons: (1) differential ACE glycosylation in the kidney (the source of ACE in the urine) compared to the lung; (2) the possible presence of ACE-binding proteins in the concentrated urine sample that may mask (shield) binding of some mAbs to urinary ACE, similar to what we previously proposed in heart tissues [72], in the spleen [73] and found in the blood (lysozyme) [40].

We speculate that a putative ACE-binding protein is present in urine that binds to the C domain to mask the binding of all mAbs to the C domain and also interferes with the binding of some mAbs to their epitopes on the N domain. To explore this hypothesis, we compared the conformational fingerprint of male and female urinary ACE before and after the depletion of proteins with MW less than 100 kD. Initially, male (#1A) and female (#5B) urine samples were concentrated using Amicon filters with 3 kD pores. Then 30X concentrated urine samples were concentrated further (10-fold) using filtration via 100 kD filters and then diluted with PBS to the initial volume. Therefore, the concentration of proteins with MW less than 100 kD was decreased 10-fold. Such depletion dramatically increased the binding of numerous mAbs with epitopes on the N and C domains of ACE to both male and female urinary ACE samples (Figure S4A,B). The changes in binding of those mAbs which were significantly decreased for urinary ACE in comparison to pure lung ACE (Figure 4C) could be attributed to the presence of relatively large ACE–binding proteins in the concentrated urine masking the N domain and the N terminal part of the C domain (the region of the 1E10 epitope). However, the binding of some mAbs (mAbs 9B9 and 5B3 on the N domain and 3F10/4C12/5G8 on the C domain) did not change (Figure S4A,B), which indicates that their epitopes were not masked by this putative ACE binding protein(s) in the human urine.

Another important functional conclusion can be made from a comparison of the effect of the depletion of 100 kD proteins on mAb binding to male and female urinary ACE (Figure S4C). The effects of 100 kD depletion on the binding of some mAbs were sex-dependent. Although 100 kD depletion affected mAbs 2H4, 2D7, 6H6, and 1E10 binding to male urinary ACE more dramatically, binding of mAbs i1A8, 3G8, 6C8, and 2H9 to female urinary ACE was more affected after 100 kD depletion. Therefore, the structural differences between male and female urinary ACE proteins that we identified (Figure 2, Figure 4A,B and Figure S2) may result in functional differences by the possible differential modulation of ACE function by putative ACE binding proteins that are present in different tissues.

The effects of depletion of 50 kD and 30 kD proteins from concentrated urine were less pronounced (Figure S5), suggesting that human albumin, which constitutes approximately 40% of all proteins in the urine [74], has a greater influence on urinary ACE conformation (and likely function) than putative ACE binding proteins with MW less than 50 or 30 kD. Therefore, it is logical to suggest that albumin, which is a natural inhibitor of ACE in the blood [75,76], also can bind to urinary (i.e., kidney) ACE and influence its function. This may be especially important in the case of proteinuria when the concentration of albumin in the urine is increased up to 100-fold during nephrotic syndrome or glomerulonephritis [77].

**Figure 5:**
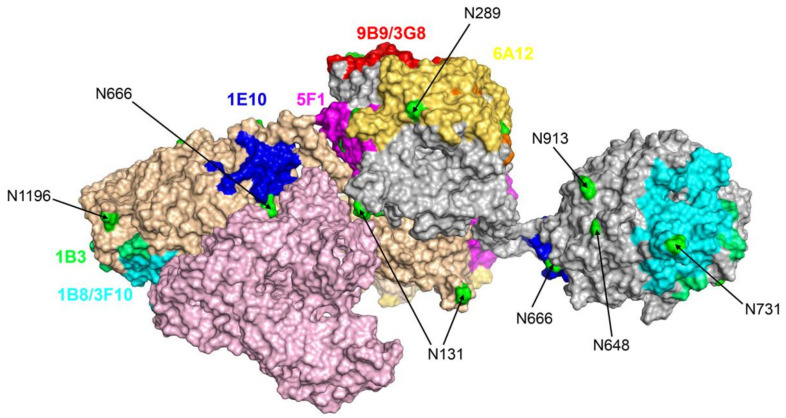
**Model of albumin docking to ACE dimer.** A model of human somatic ACE presented as a dimer [26] was used for modeling human albumin docking to ACE. The surfaces of two ACE monomers in a dimer are colored beige and grey, respectively. Albumin structure (PDB 1E78) is shown in pink. Two molecules of albumin can bind to the ACE dimer, but only one albumin molecule is shown to demonstrate the ACE epitopes interacting with albumin. The epitopes for mAbs on the N and C domains of ACE are shown in different colors. Asparagine (N) residues of the putative glycosylation sites are highlighted in lime green.

The addition of human serum albumin to ACE purified from human lung (diluted in PBS) decreased binding only for C domain epitope mAbs 1E10, 3F10, and 8H1 (Figure S6). Such an effect is similar to the effects of 100–50–30 kD depletion, or the effects of ACE isolation from different human tissues-Figure 4, S3, S4 in [72], and Figure 10 in [46], on mAb binding. When we modeled the possible binding (docking) of human albumin to two-domain ACEs (Figure 5), we observed that such docking is consistent with the dimer model of two-domain ACE [29]. In this docked model, albumin is bound primarily to the C domain (covering epitopes for mAbs 1E10 and 1B8/3F10) while also interacting with the N domain near mAbs 5F1/2D1 of the other monomer. The model matches well with changes in mAb binding to ACE in the studies cited above.

This model may have pathophysiological significance. Table S1 lists amino acids found at the interface of the ACE protein and albumin. From WES or WGS data obtained from individuals, we may predict whether putative mutations of ACE or albumin in these individuals may alter (such as decrease) the physiological inhibition of ACE by albumin [76] in the blood or kidney.

How should human urine be correctly concentrated? The first goal of this study was to establish the proper approach for the characterization of urinary ACE. Due to the very low levels of ACE in the urine (140-fold less than in serum), we elected to concentrate urine samples by 30-fold using 3 kD filter pores. This procedure increases ACE protein concentration by 30-fold, but small inhibitors present in the urine [15] will be at the same concentration as in unprocessed urine. As a result, the extent of ACE inhibition by these small inhibitors will be 30-fold less in these urine preparations. However, the concentration of albumin will also be increased in this urine by 3 kD pores, and thus it will still inhibit the activity of urinary ACE since albumin is a natural ACE inhibitor [75,76]. Indeed, our data demonstrate that the immunoreactivity of ACE towards various mAbs is different in urine concentrated using pores 3 kD compared to 100 kD (Figure S4). Therefore, to obtain structural information about urinary (kidney) ACE itself (e.g., changes in glycosylation), it is optimal to concentrate urine using filters with 100 kD pores because such an approach will dramatically decrease the effect of albumin (and other putative ACE binding proteins in human urine) on ACE activity or mAb binding to urinary ACE. In contrast, if the goal is to determine the functional parameters of urinary ACE in a more natural environment and/or estimate the effect of proteinuria on ACE functioning, it may be optimal to concentrate urine using a 3 kD pore filter to retain all possible ACE-binding proteins in the urine sample.

We also compared the immunoreactivity of urinary ACE with plasma ACE, as well as the immunoreactivity of ACE in the homogenates of human kidney and lung tissues. The mAb binding pattern observed for urinary ACE revealed substantial increases in binding to female urinary ACE for most N-domain mAbs and substantial decreases in binding for all mAbs to the C domain in comparison to female blood ACE (Figure S7A). This pattern appears very similar to the sex-specific differences in mAb binding to female and male urinary ACE protein (Figure 2A). Because serum ACE protein is much more highly sialylated than lung ACE [60] we may hypothesize that female urinary ACE is less sialylated in comparison to male urinary ACE. As a result, the binding of most mAbs to the N domain of female urinary ACE is much stronger than to the corresponding areas of male urinary ACE protein (Figure 2A,B). However, the observed decrease in binding of all mAbs to the C domain in female urinary ACE protein likely can be attributed to systemic changes in the folding of its less sialylated C domain. 

Significant differences in the conformational fingerprints of ACE in kidney homogenates compared to lung homogenates (Figure S7B) may be attributed to differential glycosylation in these two organs of certain sites in the ACE protein. These include Asn45 and Asn117 (localized in the epitopes for mAb 6H6) and Asn731 (epitope for mAb 1B8). It is a reasonable conclusion that dramatic differences in the binding of some mAbs to different types of ACE can be attributed to differential ACE glycosylation because precipitation of various types of ACE was clearly altered by different lectins. Thus, although ACE from human lung homogenates was more effectively precipitated by lectin RCA (which binds to terminal galactose residues of glycoproteins, including ACE [78]) than ACE from kidney homogenates, kidney ACE activity (as well as urinary ACE activity) was not precipitated by lectin MAA (insert in Figure S7B).

Additional support for the hypothesis that sialylation may determine sex differences in urinary ACE conformation came from the characterization of ACE derived from male volunteer #9Q. This subject demonstrated the unusual kinetic property of a lower value of ZPHL/HHL ratio in his urinary ACE (Figure 1C). We performed conformational fingerprinting of his urinary ACE compared to urinary ACE from our gold standard—subject #1A (Figure S8). Precipitation of urinary ACE from #9Q was dramatically different only for mAbs to the N domain (Figure S8A). These are essentially the same mAbs that discriminated between female and male ACE proteins in the urine (Figure 2A). Thus, urinary ACE from male #9Q exhibits similar characteristics as female urinary ACE (Figure S8B–E).

We next compared the precipitation of ACE activity from these two males by lectin RCA and by several mAbs and observed that RCA precipitation from subject #9Q is very similar to that by mAb 2D1 (which essentially does not bind male urinary ACE). Asn45 and Asn117 are in the epitope for mAb 2D1 (and 5F1) [26]. Therefore, we hypothesize that differences in sialylation of Asn45 and Asn117, as revealed by lectin RCA, determine at least in part the sex-specific differences in the conformation of urinary ACE (Figure S8F).

Western blotting for ACE protein from male (AK) and female (BK) subjects was performed with two different mAbs and demonstrated similar apparent molecular weights for male and female urinary ACE (Figure S9). These data support our hypothesis that male/female differences in ACE primarily reflect differences in the number of sialic acids rather than differences in glycan structure.

### 3.4. Urinary ACE as a Marker of Diet Efficiency?

ACE plasma levels are generally stable in individual adults [6], but blood ACE activities are significantly elevated in hyperthyroidism patients [79]. A short fasting period (12 h) did not change blood ACE [80], whereas a low-caloric diet for 8 weeks (which caused 12% of weight loss) was accompanied by an 11% decrease in serum ACE concentration [81]. Intriguingly, serum ACE activity predicted the maintenance of weight loss better than 30 other blood proteins and three steroid hormones that were analyzed [82]. Indeed, it is not realistic to expect that blood ACE could be used as a predictive marker for the maintenance of weight loss in obese patients during dieting. Therefore, attempts were made to estimate changes in urinary ACE during short periods of dieting over 24 h. The authors interpreted their data to indicate that ACE levels in the urine negatively correlated with weight loss following one day of dieting [83].

**Figure 6:**
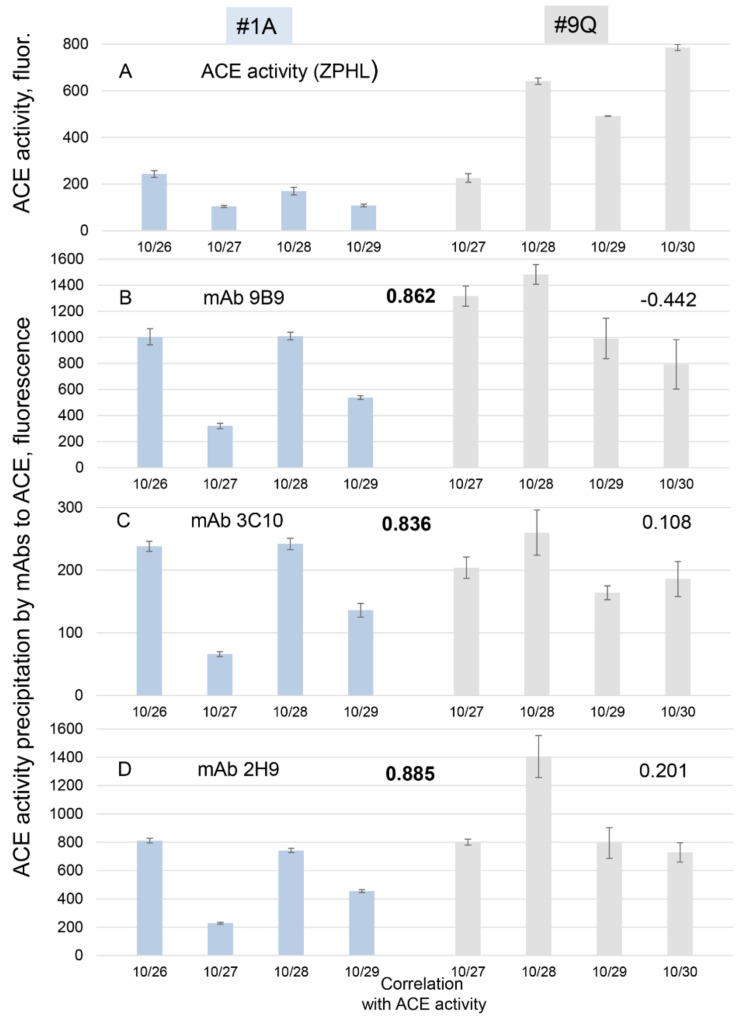
**ACE activity and the amount of ACE immunoreactive protein.** Urine (200 mL) was collected from two subjects (#1A and #9Q) on each of 4 consecutive days and stored at −20C. On the day of the experiment, urine samples were concentrated 30-fold (using filters with 3 kD pores). A total of 0.5 mL from each sample was dialyzed (using 10 kD pores), and ACE activity was determined spectrofluorometrically using substrate ZPHL (**A**). The remainder of the concentrated urine samples were used for precipitation of ACE activity by three mAbs (**B**–**D**). Correlation of ACE activity with the amount of immunoreactive ACE protein, determined with these three mAbs, was calculated for each subject.

We estimated the variability of urinary ACE phenotypes in two volunteers during 4 consecutive days of urine sampling. In contrast to blood ACE [6], urinary ACE is highly variable (Figure 6), with the daily differences in ACE activity or ACE protein varying by up to threefold. Therefore, we hypothesize that under these conditions, even short periods of dieting may influence urinary ACE activity or ACE immunoreactive protein. Thus, these urinary ACE parameters may have clinical potential as an early feedback marker on dieting and weight loss for obese individuals [83].

The amount of ACE immunoreactive protein as determined with three mAbs exhibited excellent correlation with ACE activity (r = 0.861, mean for three mAbs) for healthy volunteers (#1A). However, this correlation was practically absent (Figure 6) in volunteer #9Q, which demonstrated an abnormal ACE phenotype (Figure 1C and Figure S8). Therefore, it is optimal to determine both ACE activity and immunoreactive protein levels in order to completely characterize urinary ACE in a given individual.

### 3.5. Characterization of an ACE Phenotype Outlier (via Whole Exome Sequencing-WES)

The use of whole genome (WGS) and whole exome (WES) sequence data from multiple subjects can reveal novel mutations predicted to inactivate genes (e.g., premature stop codons), and subsequent exploration of the mechanistic effects and clinical consequences of these inactivating mutations is a valuable approach for advancing our knowledge of gene function. The combination of comprehensive ACE phenotyping as a screening format and whole exome sequencing (in some cases whole genome sequencing) of outliers may help to resolve many unclear issues of ACE biology. Prior screening studies of a large number of individuals and subsequent deep analysis of outliers for different parameters (proteins) already have produced several unexpected discoveries. Among such examples are those lowering LDL levels (*PCSK9*), decreasing susceptibility to HIV (*CCR5*), increasing exercise endurance (*ACTN3*), and increasing sepsis resistance (*CASP12*)—reviewed in [84].

In the current study, urinary ACE phenotyping of 15 apparently healthy volunteers identified at least one outlier–male subject #9Q with very low values (50% of mean) of the catalytic parameter of ACE (ZPHL/HHL ratio) (Figure 1C), as well as a “female-type” conformational fingerprint of ACE (Figure S8). The most straightforward and expected reason for these features would be a mutation in one of the ACE gene alleles. Therefore, we submitted the genomic DNA from this subject #9Q (along with DNA from the “gold standard” (subject #1A) as negative control) for WGS. However, no ACE mutations were identified in this individual (Table S2).

The next hypothesis we considered was that this subject may have a mutation in one of the known ACE binding proteins: chaperone BiP (GRP78), ribophorin 1, protein kinase C [85], albumin [76], lysozyme [40]. If such a mutation is present, then the absence or altered binding of these proteins to ACE may alter the local conformation of ACE, leading to changes in the ZPHL/HHL hydrolysis ratio. However, our sequencing data did not detect any Loss-of-Functions (LoF) mutations in any of these ACE-binding proteins.

Therefore, we are left to hypothesize that an important mutation may be present in an unknown ACE-binding protein. As an initial screen for novel ACE-binding proteins, we subtracted from the list of the mutations found in subject #9Q all the stop-codon and indels (with frameshift) mutations, as well as missense mutations with predicted damaging effects (LoF mutations), from a similar list derived from seven subjects whose WES was performed in our lab [24,86] and subject #1A (this study). The ZPHL/HHL ratios were not changed in this comparison group. This subtraction analysis allowed us to significantly decrease the number of possible candidates for novel ACE-binding proteins from 10,000 + to 400+ candidates (Table S3). However, this initial screen did not definitively identify the possible new ACE-binding protein responsible for the abnormal ACE characteristics observed in subject #9Q. Additional subjects with unchanged ZPHL/HHL ratios must be evaluated by WES and subtraction analysis for unequivocal identification of the hypothesized novel ACE binding protein.

## 4. Conclusions

Urinary samples are simple and inexpensive to obtain and represent a promising new approach for the development of precision medicine screening in ACE-related diseases. Here we demonstrate the potential power and clinical utility of urinary ACE phenotyping by defining novel sex-specific differences in urinary ACE structure and function. These variations are likely due to differential sialylation of the ACE protein, and they provide novel insights into the sex differences observed in some ACE-related diseases. Future work will build upon these insights to further define their precision medicine potential.

## Data Availability

All data are included in the manuscript.

## Supplementary Materials

The following supporting information can be downloaded at: https://www.mdpi.com/article/10.3390/biomedicines11030953/s1, Figure S1. ACE activity in human plasma; Figure S2. Gender-specific precipitation of ACE activity from male and female urine samples; Figure S3. Sex-dependence of immunoreactivity of urinary ACE; Figure S4. Effect of 100 kD depletion on immunoreactivity of urinary ACE; Figure S5. Effect of 50 kD and 30 kD depletion on immunoreactivity of urinary ACE; Figure S6. Effect of human serum albumin (HSA) on mAb binding to ACE; Figure S7. Tissue-specific precipitation of different ACEs by mAbs; Figure S8. Gender-specific precipitation of ACE activity by lectins. Figure S9. Western blotting of urinaryACE; Table S1. Analysis of the interfaces of ACE monomers and albumin; Table S2. Whole genome sequencing analysis; Table S3. Pathological mutations in the genes of subject #9Q.
