# Supplementary material for: Urinary ACE Phenotyping as a Research and Diagnostic Tool: Identification of Sex-Dependent ACE Immunoreactivity
Source: Biomedicines. 2023 Mar 20;11(3):953. doi: 10.3390/biomedicines11030953 (PMC10045976; doi:10.3390/biomedicines11030953)
Supplement: Supplementary file 1 [file biomedicines-11-00953-s001.zip › biomedicines-2267783-supplementary.pdf]

SUPPLEMENTARY MATERIALS

Urinary ACE phenotyping as a research and diagnostic tool: identification of sex-dependent ACE immunoreactivity

Alex J. Kozuch[1], Pavel A. Petukhov[2], Miklos Fagyas[3], Isolda A. Popova[4], Matthew O. Lindeblad[4], Alexander P. Bobkov[5], Armais A. Kamalov[6], Attila Toth[3], Steven M. Dudek[1], and Sergei M. Danilov[1,6]

[1]Department of Medicine, Division of Pulmonary, Critical Care, Sleep and Allergy, University of Illinois at Chicago, IL
[2]Department of Pharmaceutical Sciences, College of Pharmacy, University of Illinois at Chicago, IL
[3]Division of Clinical Physiology, Department of Cardiology, University of Debrecen, Hungary.
[4]Toxicology Research Laboratory, University of Illinois at Chicago, IL
[5]Faculty of Fundamental Medicine, Moscow University, Russia
[6]Medical Center, Moscow University, Russia

Running Head: **Urinary ACE phenotyping**

*Corresponding author:
Sergei M. Danilov, MD, PhD
Department of Medicine,
Division of Pulmonary, Critical Care, Sleep and Allergy,
University of Illinois at Chicago
CSB 915, MC 719, 840 S. Wood Ave.
Chicago, IL60612
Phone: (708) 642-0635,
E-mail: danilov@uic.edu

FIGURE LEGENDS.

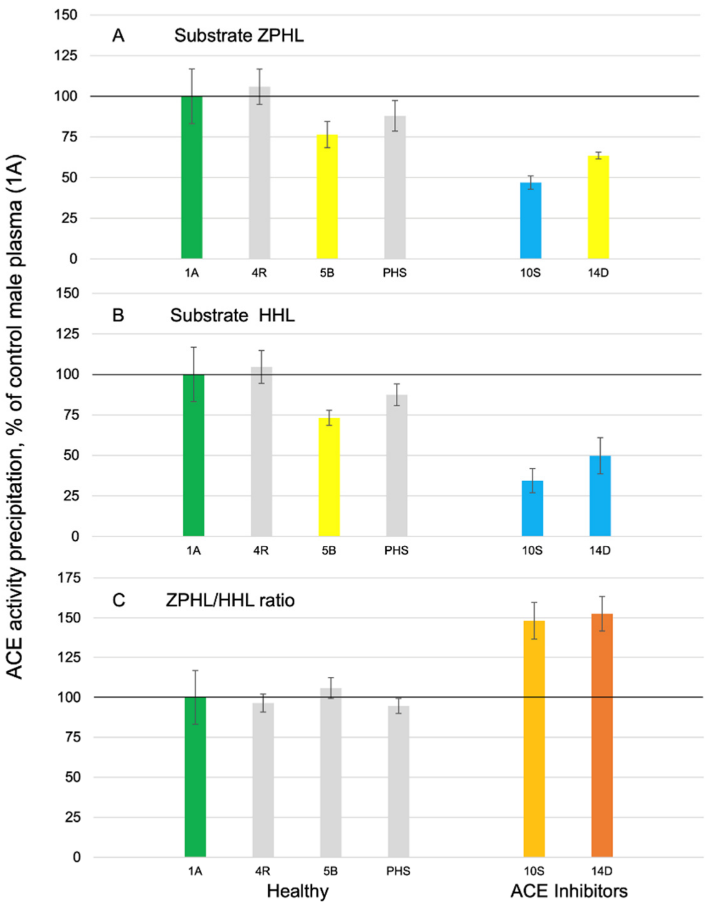

Fig. S1. ACE activity in human plasma.

ACE activity in 6 samples of 1/5 diluted plasma was quantified using a spectrofluorometric assay with Z-Phe-His-Leu (1 mM) (**A**) and Hip-His-Leu (2.5 mM) (**B**) as substrates. C. Ratio of the rate of hydrolysis of the two substrates (ZPHL/HHL ratio) in the tested samples. Data expressed as % of individual plasma ACE activity or ACE activity precipitation by mAbs of the designated "gold standard" (1A-citrated plasma of healthy 22 y.o. male-taken as 100%--green bar). Bars are colored as in Fig. 1. Mean values (+ SD) from 2-5 experiments (each made in triplicates).

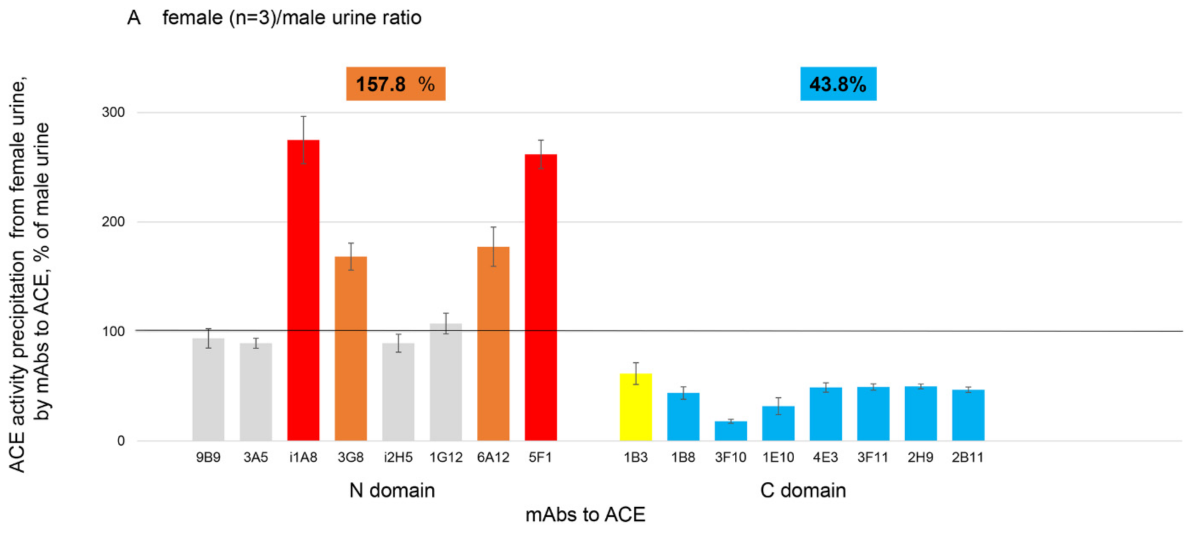

Fig. S2. Gender-specific precipitation of ACE activity from male and female urine samples. ACE activity was precipitated from 30X concentrated urine samples from 3 females and one male by 16 mAbs to N and C domains of human ACE [25]. Data are expressed as mean % of ACE activity precipitation by the given mAb from female urine and normalized by urinary ACE activity in solution to that from male urine (from 55 y.o. healthy male) used as a control sample**.** Bars are colored as in Fig. 1. Mean values (+ SD) from 2-5 experiments (each made in triplicates).

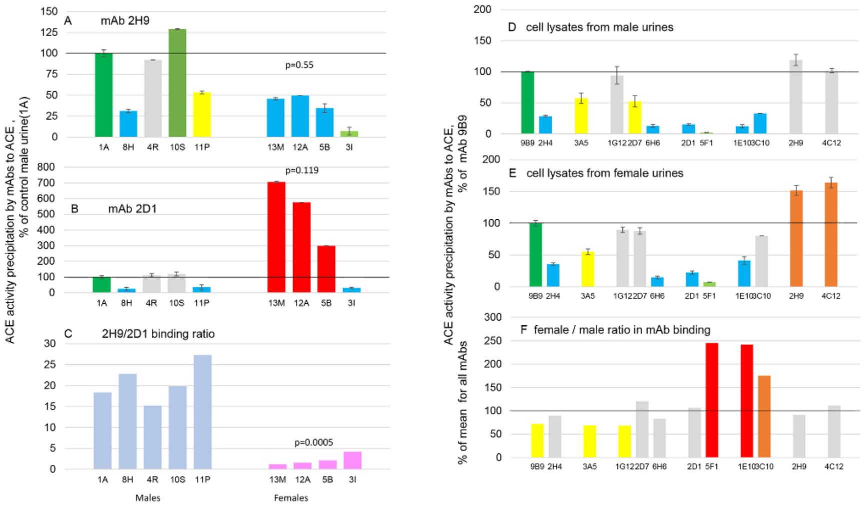

Fig. S3. Sex-dependence of immunoreactivity of urinary ACE.

A-C. ACE activity was precipitated from 30X concentrated urine samples from 5 males and 4 females by mAb 2H9 to C domain (**A**) and 2D1 –to N domain (**B**). Data are expressed as % from ACE activity (mean $\pm$ SD precipitated) from sample #1A (urine from 22 y.o. healthy male) used as a control sample**. C**. Ratio of ACE activity precipitation by mAbs 2H9 and 2D1 (2H9/2D1 binding ratio). Bars are colored as in Fig. 1. Mean values from at least 2 experiments (each made in triplicates);

D-F. ACE activity was precipitated by tested mAbs from pooled lysates of the cells sedimented from 200 ml of each of 5 male urine samples (**D**) and 4 female urine samples (**E**). Data are expressed as % from ACE activity (mean $\pm$ SD precipitated) by mAb 9B9 (green bars) used as a control mAb**. D**. Ratio of ACE activity precipitation by mAbs from female and male urine samples. Bars are colored as in Fig. 1. Mean values from at least 2 experiments (each made in triplicates). The magnitude of the bars in Fig. S2D and S2E reflect the affinity of binding of the individual mAbs to each ACE and are not as informative as the female ACE/male ACE binding ratio for lysates of the cells sedimented from urine samples (Fig. S2F), which clearly demonstrated significant differences in the local conformation between female and male membrane-bound ACE.

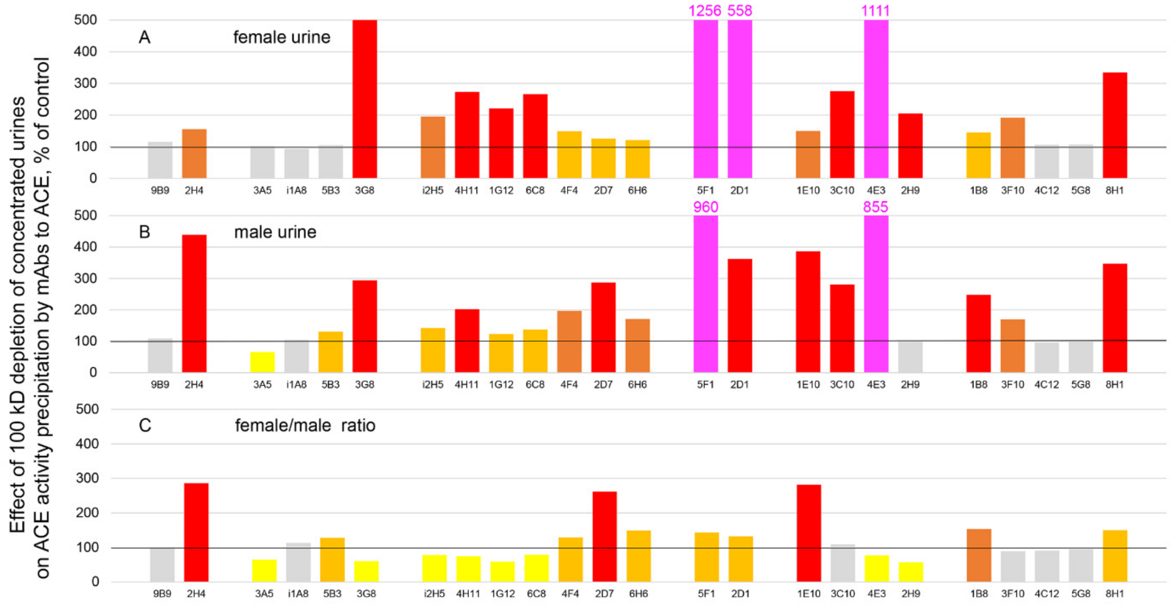

Fig. S4. Effect of 100 kD depletion on immunoreactivity of urinary ACE.

Urine samples from male (#1A) and female (#5B) (300 ml each) were concentrated 30X (3kD filters) via centrifugation. Then half of the volumes (5 ml) was subjected to additional filtration (and concentration 10-fold) via filters with 100 kD pores. These 0.5 ml of further concentrated urines were diluted to 5 ml (initial volume of 30X concentrated urine), and ACE activity was precipitated from 30X concentrated urine samples before and after 100 kD depletion. **A**-male, **B**-female. **C**. Female/male ratio of the effect of 100 kD depletion on mAb binding to ACE from urine samples. Data are expressed as % non-depleted sample. Bars are colored as in Fig. 1.

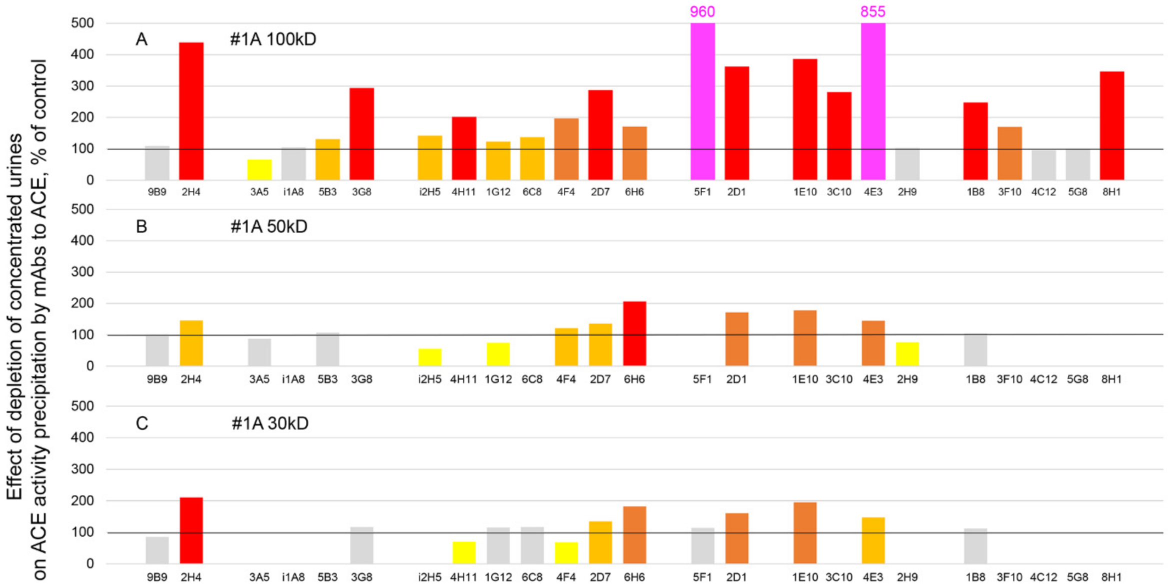

Fig. S5. Effect of 50 kD and 30 kD depletion on immunoreactivity of urinary ACE.

Depletion of 30X urine samples from male (#1A) and female (#5B) was performed as in Fig. S4, but with 50 kD and 30 kD filters. Results were presented in comparison to 100 kD depletion only for the male sample (#1A) because the pattern (effect of depletion) for female urine was similar.

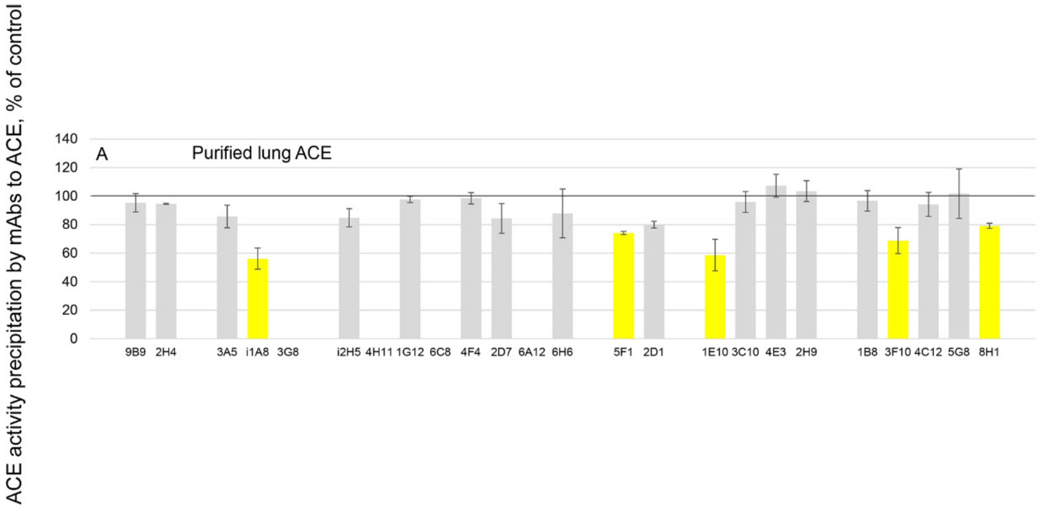

Fig. S6. Effect of human serum albumin (HSA) on mAb binding to ACE.

A. Purified human lung ACE (5-10 mU/ml) was incubated with human serum albumin (10 mg/ml –IC_{50} for ACE activity inhibition [76]) or with PBS (as negative control) for 2 hours. Then the mixture was added to plates covered with mAbs to ACE and processed as in Fig. 2. ACE activity was precipitated from treated and non-treated samples by 25 mAbs to different epitopes of human somatic ACE.

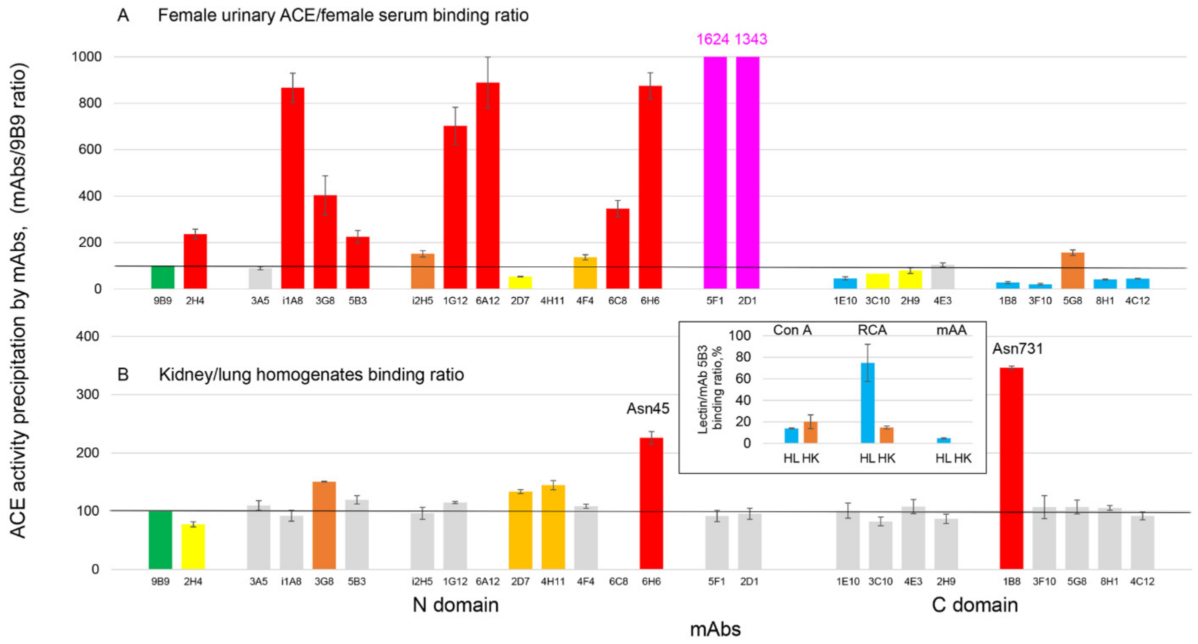

Fig. S7. Tissue-specific precipitation of different ACEs by mAbs.

A. ACE activity was precipitated by tested mAbs from 30X concentrated urine from one female (#5B), and from 1/5 diluted serum from the same female. Data are expressed as % of ACE activity (mean $\pm$ SD precipitated) from urine to that – from serum for each tested mAb**.** This ratio for mAbs that very weakly precipitate ACE from serum are shown as values (magenta bars).

B. ACE activity was precipitated by tested mAbs from human lung and kidney homogenates (1:9 weight/volume) diluted 50 times. Data are expressed as % of ACE activity (mean $\pm$ SD precipitated) from kidney to that from lung for each tested mAb**.**

Insert: ACE activity precipitation from lung and kidney homogenates by lectins expressed as % from ACE activity precipitation by mAb 5B3. Bars are colored as in Fig. 1. Mean values from at least 2 experiments (each made in triplicates).

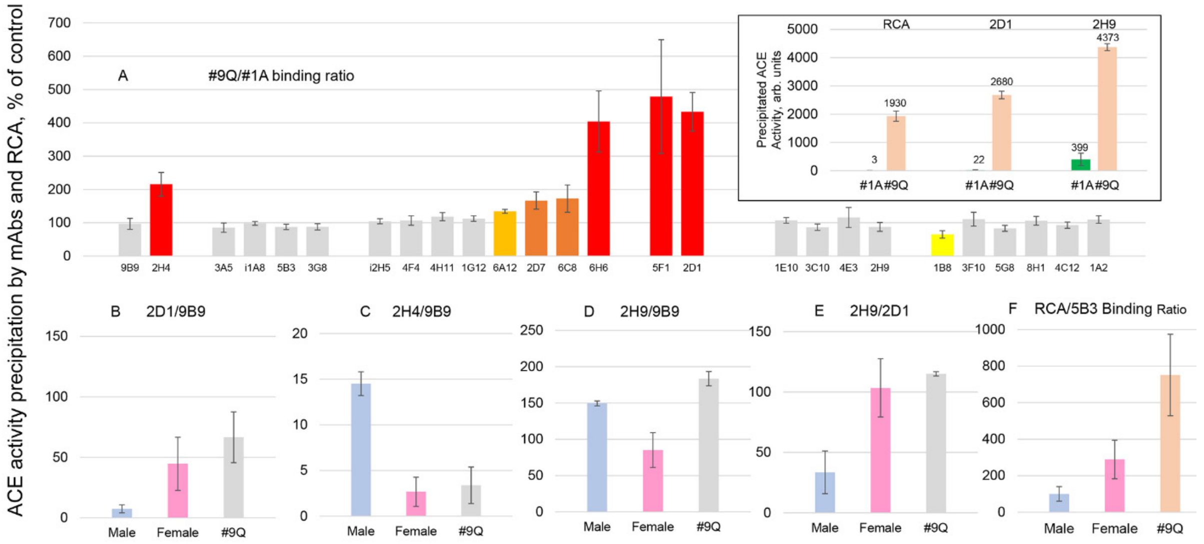

Fig. S8. Gender-specific precipitation of ACE activity by lectins.

A. ACE activity was precipitated by tested mAbs from 30X concentrated urine samples from two males (#1A and #9Q). Data are expressed as % of ACE activity (mean $\pm$ SD precipitated) from subject #9Q (36 y.o.) to that from our designated gold standard (22 y.o. healthy male (#1A) for each tested mAb**.** Bars are colored as in Fig. 1.

Insert: ACE activity precipitation from urine from these two subjects by lectins expressed as fluorescence in arbitrary units (given as values over the bars). Bars are colored as in Fig. 1. Shown are mean values from representative experiments with at least 3 replicates.

B-D. ACE activity was precipitated by several tested mAbs from urine samples of 5 males and 5 females. Data are expressed as mAb/9B9 binding ratios.

F. ACE activity precipitation from male and female samples by lectin RCA expressed as % from ACE activity precipitation by mAb 5B3. Bars are colored as in Fig. 1.

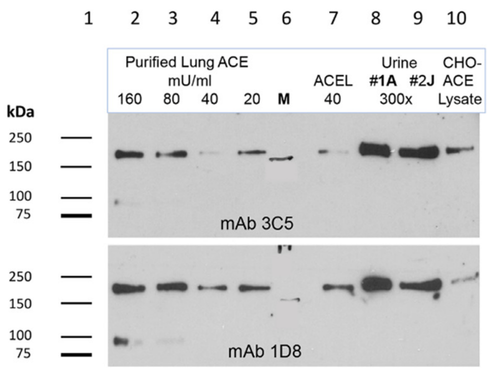

Fig. S9. Western blotting of urinary ACE.

SDS-PAGE electrophoresis was performed using 4-15% Tris-Glycine Ready Gel (Bio-Rad) Lanes: 1, 6 – Precision Dual Color Protein Ladder, lanes 2,3,4,5 – purified lung ACE - 160, 80,40, 20 U/mL respectively, lane 7 – ACE lung at 40 U/mL, lane 8, 9 – concentrated (300-fold) urine samples, male AK and female JK, respectively; Lane 10 – CHO-ACE cell lysate 1:6 dilution. After transfer, nitrocellulose was incubated with mAbs 3C5 and 1D8 [29,41].

Table S1. **Analysis of the interfaces of ACE monomers and albumin.**

The residues at the interface were determined using the “interfaceresidues.py” script in PYMOL at the cutoff of 0.25 angs^2.

**Albumin-ACE Interface Residues**	
**ACE** Chain A	**HSA**
His234-Arg-235	Ser5-Glu6
Leu244	Glu45
Gln586-Gly589	Thr52
Tyr597	Ala55-Glu60
Trp599	Asp63
Pro601-Asn606	Ser65-Leu66
Lys622	Thr76
Glu626	Thr79-Glu82
Arg629-Thr630	Cys91
Thr651-Glu652	Arg98
Lys655	Leu203-Gln204
Leu658-Lys660	Phe206-Arg209
Met662-Gln663	Lys212
Asn666	Ala229-Gln230
Lys670	Ser232-Lys233
Ala705-Glu707	Thr236-Asp237
Glu709-Glu710	Thr239-Lys240
Lys713-Ile714	Thr243-Glu244
Asp717	His247
Thr721	Asp249
Asp763-Ala765	Glu252
Arg767- Ala768	Asp256
Leu770- Phe772	Asp259
Lys775	Tyr263
	Lys317
	Ala320-Ala322
	Val335-Phe236
	Met329
**ACE** Chain B<br>**HSA** Chain C	
Asn131	His128-Asp129

Table S2. **Whole genome sequencing analysis.**

	Index Patient	Control
**Parameter**	**Pt. 9Q**	**Pt. 1K**
## genetic mutations	168088	165274
**Mutations by Type**		
nonsynonymous	10763	10722
frameshifting	215	227
stop-loss	11	13
stop-gain	84	91
synonymous	11723	11578
inframe	640	626
in noncoding RNA	1437	1366
unknown SNP	265	183
Unknown InDel	35	10
pathogenic variants	N/A	N/A
drug response variants	N/A	N/A
ACE mutations	Not found	Not found

Table S3. **Pathological mutations in the genes of subject #9Q.** (Excel file)
